# A FASII Inhibitor Prevents Staphylococcal Evasion of Daptomycin by Inhibiting Phospholipid Decoy Production

**DOI:** 10.1128/AAC.02105-18

**Published:** 2019-03-27

**Authors:** Carmen J. E. Pee, Vera Pader, Elizabeth V. K. Ledger, Andrew M. Edwards

**Affiliations:** a MRC Centre for Molecular Bacteriology and Infection, Imperial College London, London, United Kingdom

**Keywords:** AFN-1252, *Staphylococcus aureus*, antibiotic resistance, daptomycin, experimental therapeutics, phospholipids

## Abstract

Daptomycin is a treatment of last resort for serious infections caused by drug-resistant Gram-positive pathogens, such as methicillin-resistant Staphylococcus aureus. We have shown recently that S. aureus can evade daptomycin by releasing phospholipid decoys that sequester and inactivate the antibiotic, leading to treatment failure. Since phospholipid release occurs via an active process, we hypothesized that it could be inhibited, thereby increasing daptomycin efficacy. To identify opportunities for therapeutic interventions that block phospholipid release, we first determined how the host environment influences the release of phospholipids and the inactivation of daptomycin by S. aureus. The addition of certain host-associated fatty acids to the growth medium enhanced phospholipid release. However, in serum, the sequestration of fatty acids by albumin restricted their availability to S. aureus sufficiently to prevent their use in the generation of released phospholipids. This finding implies that in host tissues S. aureus may be completely dependent upon endogenous phospholipid biosynthesis to generate lipids for release, providing a target for therapeutic intervention. To test this, we exposed S. aureus to AFN-1252, an inhibitor of the staphylococcal FASII fatty acid biosynthetic pathway, together with daptomycin. AFN-1252 efficiently blocked daptomycin-induced phospholipid decoy production, even in the case of isolates resistant to AFN-1252, which prevented the inactivation of daptomycin and resulted in sustained bacterial killing. In turn, daptomycin prevented the fatty acid-dependent emergence of AFN-1252-resistant isolates *in vitro*. In summary, AFN-1252 significantly enhances daptomycin activity against S. aureus *in vitro* by blocking the production of phospholipid decoys, while daptomycin blocks the emergence of resistance to AFN-1252.

## Abstract (precis)

Daptomycin is a treatment of last resort for serious infections caused by drug-resistant Gram-positive pathogens, such as methicillin-resistant Staphylococcus aureus. We have shown recently that S. aureus can evade daptomycin by releasing phospholipid decoys that sequester and inactivate the antibiotic, leading to treatment failure.

## INTRODUCTION

Daptomycin is a lipopeptide antibiotic of last resort used to treat infections caused by drug-resistant Gram-positive pathogens, such as methicillin-resistant S. aureus (MRSA) and vancomycin-resistant enterococci (VRE) (1, 2). The target of daptomycin is the bacterial membrane, where it causes the mislocalization of enzymes required for cell wall biosynthesis, a loss of membrane potential and integrity, and rapid bacterial death (1, 3, 4).

Resistance to daptomycin can arise spontaneously via mutations in genes associated with phospholipid or peptidoglycan biosynthesis (1, 5, 6). However, while resistance has been reported to arise during treatment, it is a rare occurrence and does not explain why daptomycin treatment failure has been reported in up to 20% of cases of infective endocarditis and up to 30% of cases of complicated skin and soft tissue infection or osteomyelitis, most commonly caused by S. aureus (7, 8). Treatment failure is reduced at higher therapeutic doses of daptomycin, but host toxicity limits the concentration of the drug that can be used (1, 7, 8). In a bid to identify additional mechanisms by which S. aureus can withstand daptomycin treatment, we discovered that upon exposure to the antibiotic, S. aureus releases phospholipids into the extracellular space (9). These phospholipids act as decoys, sequestering daptomycin and preventing it from inserting into the bacterial membrane. This decoy-mediated antibiotic inactivation led to treatment failure in a murine model of invasive MRSA infection, suggesting that it could affect daptomycin efficacy in patients (9). Furthermore, the production of phospholipid decoys also occurs in enterococci and streptococci, suggesting a broadly conserved defense against membrane-acting antimicrobials (10).

The ability of released membrane phospholipids to inactivate daptomycin can be compromised in S. aureus by the quorum-sensing-triggered production of small cytolytic peptides known as the alpha phenol-soluble modulins (PSMα) (9). These peptides appear to compete with daptomycin for the phospholipid and thereby prevent inactivation of the antibiotic (9). While this may appear paradoxical, many invasive infections are caused by S. aureus strains defective for PSMα production due to defects in the accessory gene regulator (Agr) quorum-sensing system that triggers expression of the peptides (11–13). Furthermore, as serum apolipoproteins inhibit the Agr system and sequester PSMs, wild-type (WT) bacteria would be expected to inactivate daptomycin in the bloodstream (14–17).

The mechanism by which daptomycin triggers phospholipid release is currently undefined. However, we have shown that it is an active process that requires energy, as well as protein, cell wall, and lipid biosynthesis (9, 10). The requirement for fatty acid biosynthesis for phospholipid release is important because it raises the prospect of targeting this process to enhance daptomycin efficacy. We have shown previously that inhibition of the FabF component of the fatty acid synthesis type II (FASII) pathway, using the antibiotic platensimycin, completely blocked phospholipid release (9, 10). While platensimycin is unsuitable as a therapeutic drug due to poor pharmacological properties, the FabI inhibitor AFN-1252 shows more promising characteristics, and a prodrug variant is currently undergoing phase 2 clinical trials (18, 19). However, despite excellent *in vitro* activity, the therapeutic value of FASII inhibitors as monotherapeutic agents has attracted much debate (20, 21). Several bacteria, including S. aureus, can utilize fatty acids present in the host to generate phospholipids (21–24). Although wild-type S. aureus strains cannot fully substitute endogenous fatty acids for exogenous fatty acids synthesized via FASII, there is evidence that some clinical isolates (up to 7%) have acquired mutations that enable them to fully or partially bypass endogenous fatty acid biosynthesis by utilizing host-derived fatty acids (22, 25, 26). Furthermore, *in vitro* experimentation suggests that the acquisition of such mutations is dependent upon the presence of host-associated fatty acids, which means that the frequency at which resistance to AFN-1252 emerges *in vivo* may have been underestimated (25, 26). As such, the long-term viability of fatty acid synthesis inhibitors, such as AFN-1252, as monotherapeutic antibacterial drugs is unclear, and their ability to block daptomycin-induced phospholipid release in the presence of exogenous fatty acids is undetermined (20, 21).

Therefore, the aims of this work were to understand how the availability of fatty acids in the host influences the production of phospholipid decoys and determine whether AFN-1252 can be used in combination with daptomycin to provide a viable approach to combating MRSA infection.

## RESULTS

### Exogenous fatty acids modulate daptomycin-induced phospholipid release.

Since S. aureus can incorporate exogenous fatty acids into membrane phospholipid production, it was hypothesized that host-derived fatty acids would contribute to the production of lipids required for daptomycin-induced phospholipid release (21–24).

To enable accurate measurements of phospholipid release, these experiments were done in tryptic soy broth (TSB) containing, or not, one of several different fatty acids found in normal human serum (27). To avoid the Agr system compromising daptomycin inactivation, these initial experiments employed the S. aureus USA300 LAC (USA300) Δ*agrA* mutant (Table 1), which has the same daptomycin MIC as the wild type (Table 2) (9).

**TABLE 1:** Strains used in this study

Strain	Relevant characteristics/information	Agr activity (hemolytic activity)	Reference or source
USA300 LAC	Wild-type community-associated MRSA strain isolated	+++	43
USA300 LAC Δ*agrA*	Agr-defective mutant lacking the *agrA* gene	−	9
CC6	MRSA isolated from a bloodstream infection	+++	CHX^*a*^
CC7	MRSA isolated from a bloodstream infection	−/+	CHX
CC9	MRSA isolated from a bloodstream infection	−/+	CHX
CD1	MRSA isolated from a bloodstream infection	−	CHX
CD2	MRSA isolated from a bloodstream infection	++	CHX
CD3	MRSA isolated from a bloodstream infection	−	CHX
CD4	MRSA isolated from a bloodstream infection	+++	CHX
CD5	MRSA isolated from a bloodstream infection	−	CHX
CD6	MRSA isolated from a bloodstream infection	−	CHX
CD8	MRSA isolated from a bloodstream infection	−/+	CHX

a CHX, Charing Cross Hospital Clinical Diagnostic Microbiology Laboratory.

**TABLE 2:** MICs of daptomycin, AFN-1252, and oxacillin in relevant growth media

Antibiotic and medium^*a*^	MIC (μg ml^−1^)											
	USA300 WT	USA300 Δ*agrA*	CC6	CC7	CC9	CD1	CD2	CD3	CD4	CD5	CD6	CD8
Daptomycin												
TSB^0.5Ca^	1	1	1	1	1	2	2	1	1	1	1	1
TSB^1.25Ca^	0.5	0.5	0.25	0.25	0.25	0.5	0.5	0.25	0.25	0.5	0.25	0.25
MHB^1.25Ca^	0.25	0.25	0.25	0.25	0.25	0.25	0. 5	0.25	0.25	0.25	0.25	0.25
TSB/serum	0.5	0.5	0.5	0.5	0.5	1	1	0.5	0.5	0.5	0.5	0.5
AFN-1252												
TSB^0.5Ca^	0.015	0.015	0.015	0.008	0.015	0.015	0.015	0.015	0.015	0.015	0.015	0.008
TSB^1.25Ca^	0.015	0.015	0.015	0.008	0.03	0.015	0.03	0.015	0.03	0.015	0.015	0.008
MHB^1.25Ca^	0.015	0.015	0.015	0.008	0.015	0.015	0.015	0.008	0.015	0.008	0.008	0.008
TSB/serum	0.06	0.06	0.06	0.06	0.06	0.06	0.06	0.06	0.06	0.06	0.06	0.06
Oxacillin												
TSB^0.5Ca^	4	4	32	8	4	>128	32	128	32	16	16	16
TSB^1.25Ca^	4	4	8	8	4	>128	8	128	16	8	8	8
MHB^1.25Ca^	2	2	8	2	2	>128	4	128	8	4	2	2
TSB/serum	8	8	64	16	8	>128	32	128	32	32	32	32

a TSB^0.5Ca^, TSB containing 0.5 mM CaCl_2_; TSB^1.25Ca^, TSB containing 1.25 mM CaCl_2_; MHB^1.25Ca^, Mueller-Hinton broth containing 1.25 mM CaCl_2_; TSB/serum, TSB containing 1.25 mM CaCl_2_ and 50% normal human serum.

**FIG 1:**
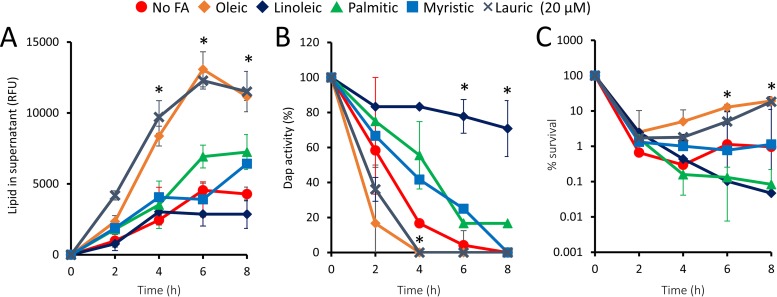
Effect of exogenous fatty acids on daptomycin (Dap)-induced phospholipid release, daptomycin inactivation, and bacterial survival. S. aureus Δ*agrA* was exposed to daptomycin (20 μg ml^−1^) in the presence of the indicated fatty acid supplements (20 μM) or no fatty acid (No FA), and the release of phospholipids (A), antibiotic activity (B), and bacterial survival (C) were measured over time. Data represent the means from 4 independent experiments, and error bars show the standard deviation of the mean. Values significantly different (*P* < 0.05) from those for bacteria in broth without fatty acid supplements were identified by 2-way repeated-measures analysis of variance (ANOVA) and Dunnett’s *post hoc* test (*).

Exposure of the S. aureus USA300 Δ*agrA* mutant to daptomycin in the absence of exogenous fatty acids resulted in the release of phospholipids into the extracellular space (Fig. 1A). Supplementation of the TSB growth medium with linoleic acid had no effect on the rate or quantity of phospholipid released, while the presence of myristic or palmitic acid resulted in a small increase in the quantity of phospholipids released at the latest time point (Fig. 1A). In contrast, the presence of oleic or lauric acid significantly enhanced both the rate and the quantity of phospholipids released relative to those seen in TSB without fatty acids (Fig. 1A).

The increased release of phospholipids from bacteria incubated with oleic or lauric acid resulted in a slightly higher rate of daptomycin inactivation, while the presence of linoleic, palmitic, or myristic acid reduced the rate of daptomycin inactivation (Fig. 1B). Of note, S. aureus failed to fully inactivate daptomycin in the presence of palmitic or linoleic acid, indicating that exogenous fatty acids can retard as well as promote the rate of phospholipid-mediated daptomycin inactivation (Fig. 1B).

In keeping with the effect of individual fatty acids on daptomycin inactivation, the presence of oleic or lauric acid promoted bacterial survival to a rate 10-fold above that seen for S. aureus incubated without fatty acids by 8 h. In contrast, the presence of palmitic or linoleic acid reduced the rate of survival approximately 10-fold, while myristic acid had no effect (Fig. 1C).

**FIG 2:**
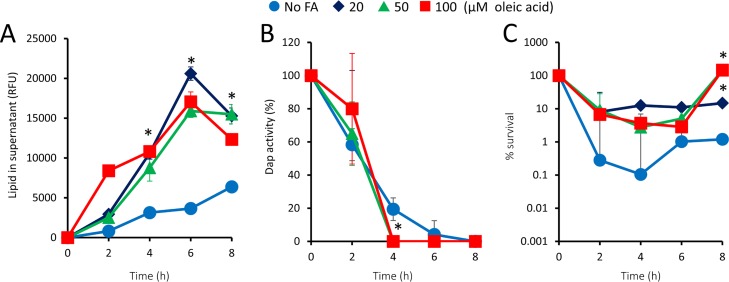
Effect of increasing concentrations of oleic acid on daptomycin-induced phospholipid release, daptomycin inactivation, and bacterial survival. The S. aureus Δ*agrA* mutant was exposed to daptomycin (20 μg ml^−1^) in the presence of the indicated concentrations of oleic acid, and the release of phospholipids (A), antibiotic activity (B), and bacterial survival (C) were measured over time. For panel B, the values for 20 μM are obscured by the symbols representing 100 μM. Data represent the means from 4 independent experiments, and error bars show the standard deviation of the mean. Values significantly different (*P* < 0.05) from those for bacteria in broth without fatty acid supplements were identified by 2-way repeated-measures ANOVA and Dunnett’s *post hoc* test (*).

Next, we determined how the concentration of exogenous fatty acid affected phospholipid release and bacterial survival. As shown in Fig. 1A, the presence of 20 μM oleic acid promoted phospholipid release in response to daptomycin challenge (Fig. 2A). However, increasing the concentration of oleic acid up to 100 μM (which is similar to that found in serum [27]) did not increase the level of phospholipid release above that seen with 20 μM (Fig. 2A). In keeping with this, the presence of 100 μM oleic acid did not significantly affect the rate of daptomycin inactivation relative to that seen with 20 μM, nor did the higher concentration of the fatty acid reduce the initial rate of daptomycin-mediated killing (Fig. 2B and C). However, the highest concentrations of oleic acid did promote the rate of recovery once daptomycin was inactivated, presumably by providing precursors to the energetically expensive process of membrane biogenesis (Fig. 2B and C).

### Serum albumin restricts the utilization of oleic acid by S. aureus for phospholipid release.

**FIG 3:**
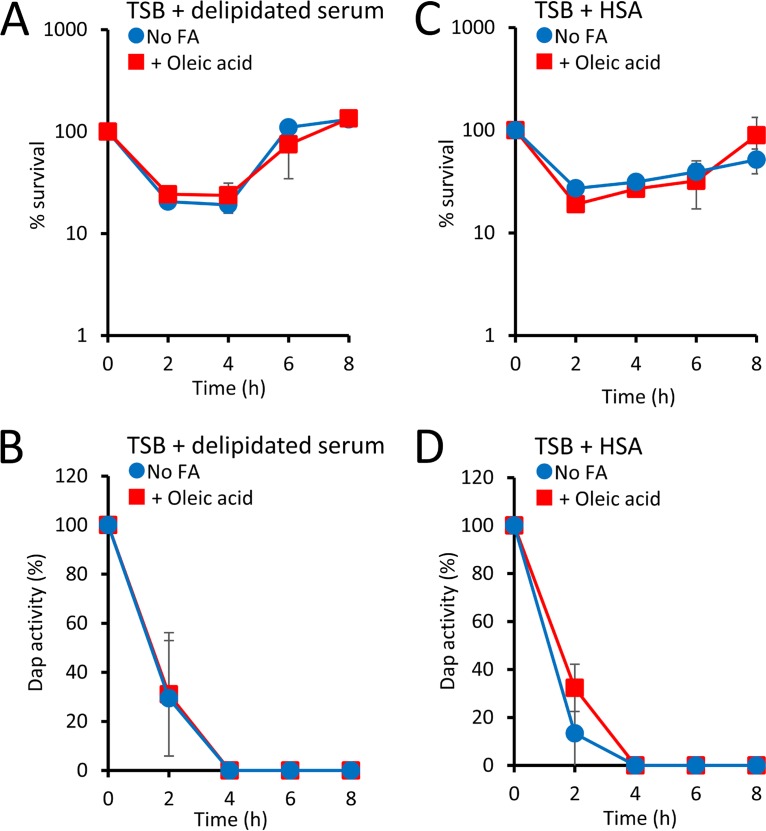
Human serum albumin prevents the use of exogenous oleic acid in daptomycin-induced phospholipid release. (A, B) The S. aureus Δ*agrA* mutant was exposed to daptomycin (20 μg ml^−1^) in TSB containing 50% delipidated human serum containing oleic acid (20 μM) or not (No FA), and bacterial survival (A) and antibiotic activity (B) were measured over time. (C, D) In a similar experiment, the S. aureus Δ*agrA* mutant was exposed to daptomycin in TSB containing human serum albumin (HSA) and supplemented with oleic acid (20 μM) or not (No FA), and bacterial survival (C) and antibiotic activity (D) were measured over time. Data represent the means from 4 independent experiments, and error bars show the standard deviation of the mean. There were no significant differences in values obtained with oleic acid compared to those obtained in unsupplemented medium (*P* > 0.05), as determined by 2-way repeated-measures ANOVA.

Having established that fatty acids can modulate phospholipid release in TSB, we wanted to determine whether their presence in the host context had a similar effect. To do this, we first supplemented TSB with 50% delipidated human serum, which is deficient for fatty acids. Similar to what was seen in TSB alone, exposure of the Δ*agrA* mutant to daptomycin in TSB containing 50% delipidated human serum resulted in an initial fall in the CFU counts, followed by a period of recovery (Fig. 3A). However, in contrast to our observations for TSB (Fig. 1C), the addition of oleic acid to TSB containing 50% delipidated serum had no effect on bacterial survival (Fig. 3A). In keeping with these data, the presence of oleic acid had no effect on the rate at which the bacteria inactivated daptomycin (Fig. 3B). This indicated that the ability of S. aureus to use oleic acid to promote phospholipid release was restricted by a factor found in serum but not TSB, although this was not quantified directly, as serum components interfered with the dye-based assay system.

Fatty acids present in the bloodstream are typically bound to serum albumin, which acts as a carrier protein (28). To determine whether the presence of this host protein restricted the availability of oleic acid for use in phospholipid release-mediated inactivation of daptomycin, the S. aureus Δ*agrA* mutant was exposed to daptomycin in TSB containing oleic acid and human serum albumin (HSA). In contrast to the findings with TSB only, the presence of HSA completely abrogated the increased rate of daptomycin inactivation and bacterial survival observed on supplementation with oleic acid, presumably due to sequestration of the fatty acid by the protein (Fig. 3C and D).

### AFN-1252 blocks daptomycin-induced phospholipid release in the presence of unbound oleic acid.

The finding that HSA prevented the use of exogenous oleic acid by S. aureus to promote the rate of daptomycin inactivation indicated that this process is likely to be entirely dependent upon the FASII pathway *in vivo*. Therefore, we hypothesized that the FASII inhibitor AFN-1252 would enhance daptomycin activity against S. aureus by blocking the production of phospholipid decoys.

**FIG 4:**
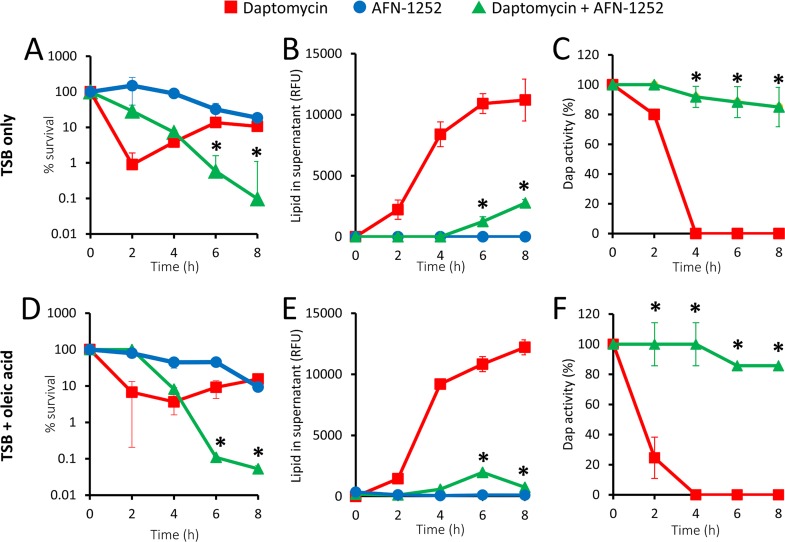
AFN-1252 blocks phospholipid release and therefore preserves daptomycin activity. The S. aureus Δ*agrA* mutant was exposed to daptomycin (20 μg ml^−1^), AFN-1252 (0.15 μg ml^−1^), or both antibiotics in the absence (A, B, C) or presence (D, E, F) of oleic acid (20 μM). During incubation, bacterial survival (A, D), the quantity of phospholipid released into the supernatant (B, E), and daptomycin activity (C, F) were measured over 8 h. Data represent the means from 4 independent experiments, and error bars show the standard deviation of the mean. Values significantly different (*P* < 0.05) from those obtained with bacteria exposed to daptomycin only were identified by 2-way repeated-measures ANOVA and Dunnett’s *post hoc* test (*).

Alone, AFN-1252 (0.15 μg ml^−1^) showed bacteriostatic activity (a <10-fold drop in CFU counts after 8 h) (Fig. 4A). As described previously, the CFU counts of the S. aureus Δ*agrA* mutant exposed to daptomycin fell initially, before recovering due to the release of phospholipids that led to the inactivation of the antibiotic (Fig. 4A to C) (9). However, when the S. aureus Δ*agrA* mutant was exposed to daptomycin in the presence of AFN-1252, there was a >500-fold drop in CFU counts, with no recovery of the bacterial population (Fig. 4A). Further analysis revealed that AFN-1252 almost completely blocked daptomycin-induced phospholipid release and the associated daptomycin inactivation (Fig. 4B and C), providing an explanation for the synergy observed when these antibiotics were used in combination.

While our data indicated that HSA restricts the utilization of exogenous fatty acids for phospholipid release (Fig. 3C and D), we considered the possibility that some unbound lipids may arise during infection because of damage to host tissues. Therefore, we repeated the experiments whose results are described in Fig. 4A to C in the presence of oleic acid without HSA, since this lipid was previously shown to significantly promote phospholipid release (Fig. 1A). The data generated from these experiments were almost identical to those from experiments done in the absence of the fatty acid (Fig. 4D to F). AFN-1252 showed clear synergistic activity when used in combination with daptomycin by blocking phospholipid release, even in the presence of unbound oleic acid (Fig. 4E). This resulted in the maintenance of daptomycin activity and a sustained killing effect on S. aureus (Fig. 4D and F). Together, these data demonstrate that AFN-1252 prevents the production of phospholipid decoys, even in the presence of exogenous fatty acids which would otherwise enhance phospholipid release.

### AFN-1252 blocks phospholipid release triggered by a range of daptomycin concentrations.

The bactericidal activity of daptomycin is dependent upon the concentration of both the antibiotic and calcium ions (1). To determine how these factors affected the inhibition of phospholipid release by AFN-1252 and the consequences for bacterial survival, both wild-type (WT) and Δ*agrA* mutant S. aureus strains were exposed to various concentrations of daptomycin in broth supplemented with 0.5 mM or 1.25 mM CaCl_2_ in the presence or absence of the FASII inhibitor (0.15 μg ml^−1^).

**FIG 5:**
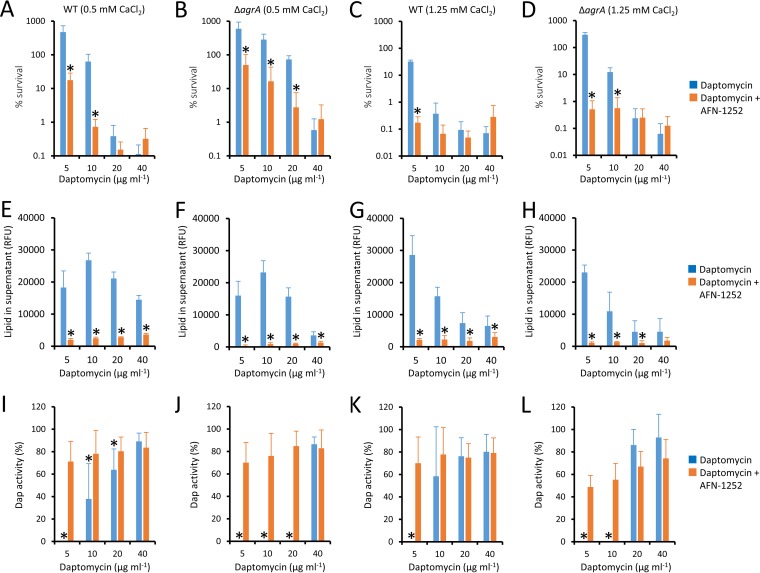
AFN-1252 blocks phospholipid release at various concentrations of daptomycin. Wild-type (WT) S. aureus (A, C, E, G, I, K) or the Δ*agrA* mutant (B, D, F, H, J, L) was exposed to daptomycin at the indicated concentrations in the absence (blue bars) or presence (orange bars) of AFN-1252 (0.15 μg ml^−1^) in the presence of 0.5 mM CaCl_2_ (A, B, E, F, I, J) or 1.25 mM CaCl_2_ (C, D, G, H, K, L). After 8 h of incubation, bacterial survival (A to D), phospholipid release (E to H), and daptomycin activity (I to L) were measured. Values from experiments done with AFN-1252 significantly different (*P* < 0.05) from those obtained with bacteria exposed to daptomycin only were identified by a paired Student's *t* test (*).

Daptomycin caused the dose-dependent killing of both WT and Δ*agrA* mutant S. aureus strains, which was greater at 1.25 mM than 0.5 mM CaCl_2_, with a >1,000-fold reduction in CFU counts being seen at 40 μg ml^−1^ of the antibiotic (Fig. 5A to D) (1, 10). As expected from our earlier studies, at both CaCl_2_ concentrations, the survival of the Δ*agrA* mutant was greater than that of the WT at lower concentrations of daptomycin, but killing was similar between the strains at the highest concentration of the antibiotic tested (40 μg ml^−1^) (Fig. 5A to D) (9). At lower concentrations of daptomycin, the presence of AFN-1252 reduced bacterial survival by ∼10- to 100-fold but had no effect on bacterial survival in the presence of the highest concentration of the lipopeptide antibiotic tested (Fig. 5A to D).

As observed previously, phospholipid release was generally greater at lower concentrations of daptomycin (10) and reduced in the presence of the higher concentration of calcium (Fig. 5E to H). However, regardless of the experimental conditions or the quantity of phospholipids released, the presence of AFN-1252 significantly reduced phospholipid release from the WT or Δ*agrA* mutant S. aureus strain to almost undetectable levels (Fig. 5E to H).

In agreement with previous work, the Δ*agrA* mutant was significantly more efficient than the WT at inactivating daptomycin (Fig. 5I to L) (9). In the presence of 0.5 mM CaCl_2_, WT S. aureus could only partially inactivate 10 μg ml^−1^ daptomycin, whereas the Δ*agrA* mutant was able to completely inactivate the lipopeptide at 20 μg ml^−1^ (Fig. 5I and J). At 1.25 mM CaCl_2_, WT S. aureus fully inactivated daptomycin at 5 μg ml^−1^, but the Δ*agrA* mutant inactivated the antibiotic at 10 μg ml^−1^ (Fig. 5K and L). However, for both the WT and the Δ*agrA* mutant, the presence of AFN-1252 prevented the inactivation of daptomycin, in keeping with the ability of this antibiotic to prevent phospholipid release (Fig. 5I to L) (9, 10).

In summary, at concentrations of daptomycin that are inactivated by released phospholipids, AFN-1252 promotes bacterial killing. However, at concentrations of daptomycin that cannot be inactivated by S. aureus, AFN-1252 has little or no effect on bacterial survival. This provides additional evidence that the FASII inhibitor synergizes with the lipopeptide antibiotic by blocking the release of phospholipids that inactivate daptomycin.

### AFN-1252 blocks daptomycin-induced phospholipid release in human serum.

To further explore how the host environment might influence daptomycin-induced phospholipid release and whether AFN-1252 would be expected to block this, we used TSB containing 50% normal human serum. In addition to providing fatty acids in their natural state and concentration, this system also accounts for the effects of antibiotic binding to serum proteins and the suppression of Agr activity by apolipoproteins.

**FIG 6:**
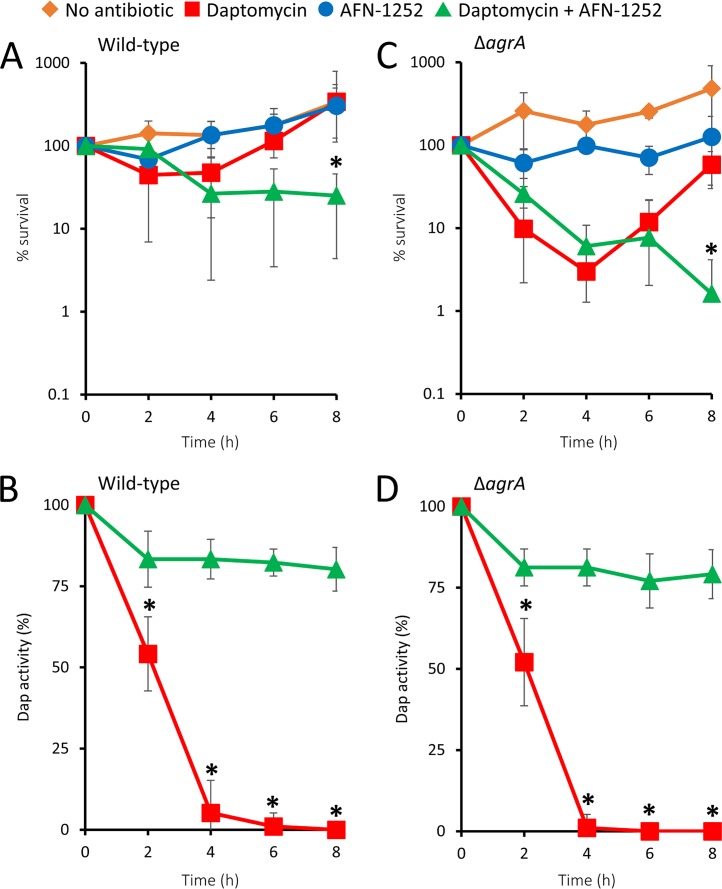
AFN-1252 preserves daptomycin activity in serum. Wild-type S. aureus USA300 or the Δ*agrA* mutant was exposed to daptomycin (20 μg ml^−1^), AFN-1252 (0.15 μg ml^−1^), both antibiotics, or neither antibiotic in TSB containing 50% normal human serum. During incubation, bacterial survival (A, C) and daptomycin activity (B, D) were measured over 8 h. Data represent the means from 4 independent experiments, and error bars show the standard deviation of the mean. Values significantly different (*P* < 0.05) from those obtained with bacteria exposed to daptomycin only were identified by 2-way repeated-measures ANOVA and Dunnett’s *post hoc* test (*).

As reported earlier, the presence of serum resulted in slightly increased MICs of some strains for both daptomycin and AFN-1252, due to the binding of the antibiotics by serum proteins (Table 2) (29, 30). Exposure of wild-type strain S. aureus USA300 to daptomycin alone resulted in a brief decline in the CFU counts over the first 2 h, followed by an increase in bacterial numbers (Fig. 6A). Unfortunately, the high lipid content of serum prevented accurate measurement of phospholipid release. However, bacterial survival correlated well with the inactivation of daptomycin, which occurred within 4 h (Fig. 6B). A broadly similar survival profile was seen for the Δ*agrA* mutant, suggesting that the presence of serum negates previously reported differences in daptomycin inactivation mediated by Agr (Fig. 6C and D) (9).

Despite the increased MIC for AFN-1252 in serum, the presence of the FASII inhibitor prevented daptomycin inactivation by both wild-type S. aureus and the Δ*agrA* mutant, resulting in increased bacterial killing over the duration of the assay (Fig. 6A to D).

### AFN-1252 blocks daptomycin-induced phospholipid release by clinical isolates.

To test whether daptomycin-induced phospholipid release is a common property of clinical MRSA isolates and whether it is blocked in these strains by AFN-1252, we examined a panel of 10 MRSA isolates from bloodstream infections. In keeping with previous reports, some of these isolates were hemolytic, while others were not, indicative of a loss of Agr activity (Table 1) (11).

**FIG 7:**
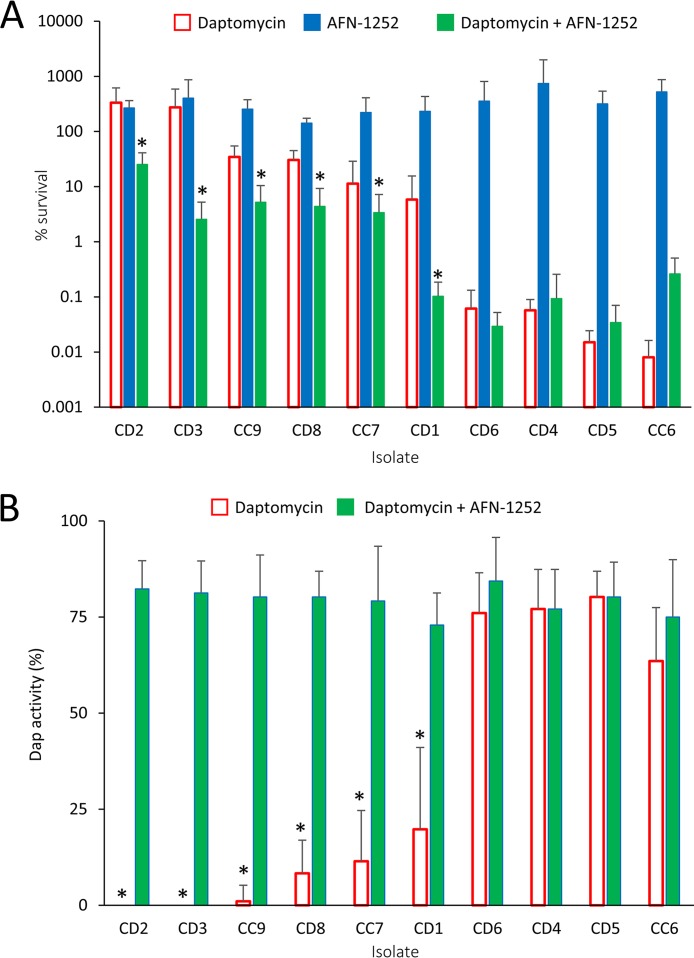
AFN-1252 prevents daptomycin inactivation by clinical MRSA isolates. Clinical MRSA isolates from bloodstream infections were exposed to daptomycin (20 μg ml^−1^), AFN-1252 (0.15 μg ml^−1^), or both antibiotics in TSB containing 50% normal human serum. After 8 h of incubation, bacterial survival (A) and daptomycin activity (B) were measured. Data represent the means from 4 independent experiments, and error bars show the standard deviation of the mean. Values significantly different (*P* <0.05) from those obtained with bacteria exposed to daptomycin only were identified by a paired Student's *t* test (*).

Exposure of each of the 10 isolates to daptomycin in the presence of normal human serum resulted in a wide variation in survival levels, with the CFU counts of some strains increasing slightly but those of others declining >10,000-fold after 8 h of challenge, which was independent of their Agr activity (Fig. 7A; Table 1). Measurement of daptomycin activity at the end of the experiment revealed that 6 strains had inactivated daptomycin fully or by at least 80%, while the other 4 strains did not significantly reduce the activity of the lipopeptide antibiotic (Fig. 7B). Of note, all 6 of the isolates that fully or partially inactivated daptomycin survived at higher levels (>5% survival) than the 4 isolates that did not reduce the activity of the antibiotic (<0.07% survival). There was no correlation between the oxacillin MIC and the ability of an isolate to inactivate daptomycin (Table 2).

In keeping with the findings of our experiments with the USA300 strain, the presence of AFN-1252 blocked the inactivation of daptomycin, which correlated with a significant reduction in the survival of the daptomycin-inactivating bacterial isolates (Fig. 7A and B). In contrast, AFN-1252 did not significantly affect the survival of bacteria that did not inactivate daptomycin, providing additional evidence that AFN-1252 promotes daptomycin’s bactericidal activity by preventing S. aureus from releasing phospholipid decoys that enable the bacterium to evade the lipopeptide antibiotic (Fig. 7A and B).

### Exogenous fatty acids enable emergence of resistance to AFN-1252.

The data described above indicated that use of the FASII inhibitor AFN-1252 in combination with daptomycin may be a promising therapeutic approach. To determine the propensity of S. aureus to acquire spontaneous resistance to AFN-1252, 10 parallel cultures of the USA300 Δ*agrA* mutant were repeatedly challenged with AFN-1252 (0.15 μg ml^−1^) in the absence or presence of a physiologically relevant fatty acid cocktail as described previously (26). Given the impact of HSA on fatty acid sequestration, parallel assays were done with or without the serum protein. After each exposure, bacterial susceptibility to AFN-1252 was determined by broth microdilution assays to establish the MIC.

**FIG 8:**
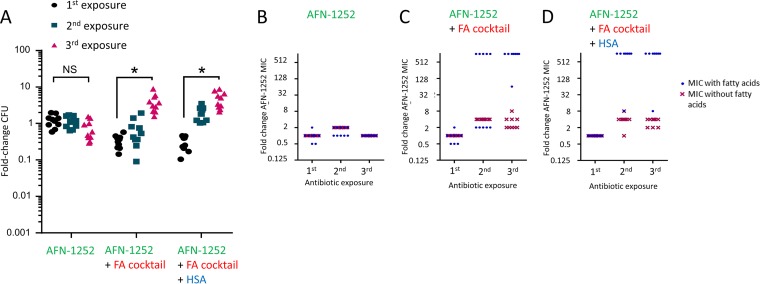
Exogenous fatty acids enable the acquisition of resistance to AFN-1252. Ten parallel cultures of the S. aureus Δ*agrA* mutant were exposed to 3 rounds of AFN-1252 (0.15 μg ml^−1^) treatment in the absence or presence of 50 μM fatty acid (FA) cocktail and the absence or presence of human serum albumin (HSA) for 8 h before bacterial replication (A) and the AFN-1252 MIC (B, C, D) were determined in the absence or presence of the fatty acid cocktail. Each symbol represents an independent culture (*n* = 10 in each case). Differences in survival between the 1st and 3rd rounds of AFN-1252 exposure under identical conditions were analyzed using a one-way ANOVA with Dunn’s multiple-comparison test (*, *P* < 0.001).

As expected from a previous report, there was very little change in bacterial growth (Fig. 8A) or the MIC (Fig. 8B) when S. aureus was repeatedly exposed to AFN-1252 in the absence of fatty acids (26). However, in keeping with previous work, by the third round of exposure to AFN-1252 in the presence of fatty acids, with or without HSA, S. aureus was able to replicate in the presence of the antibiotic (Fig. 8A) (26). The ability of S. aureus to grow in the presence of AFN-1252 after repeated exposure to the antibiotic in the presence of fatty acids, regardless of the presence of HSA, correlated well with data from subsequent MIC assays (Fig. 8C and D). When fatty acids were included in the MIC assays, there was a significant and large increase in the MICs of most cultures from 0.03125 μg ml^−1^ to more than 16 μg ml^−1^ (>512-fold) for bacteria that were exposed to AFN-1252 in the presence of exogenous fatty acids (Fig. 8C and D). Since fatty acid-dependent AFN-1252 resistance has been most commonly linked to mutations in the *fabD* gene (26), we examined this locus in two randomly selected AFN-1252-resistant isolates from this assay. This revealed an 826G>T substitution, which corresponds to FabD G276STOP, resulting in a truncated protein in one isolate, while the other had a 3G>A substitution, which would be expected to result in failure of the ribosome to recognize the ATG start codon, resulting in a lack of FabD production.

Together, these data confirm previous work showing that repeated exposure of S. aureus to AFN-1252 in the presence of exogenous fatty acids facilitated the emergence of fatty acid-dependent resistance to this antibiotic, at least in part via mutations in the *fabD* gene (26).

### Daptomycin prevents fatty acid-dependent emergence of resistance to AFN-1252.

**FIG 9:**
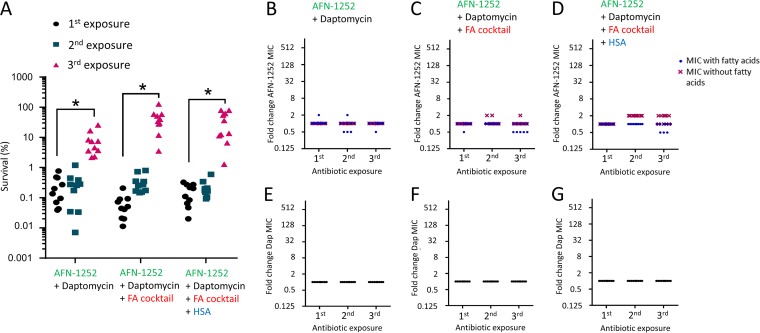
Daptomycin prevents the acquisition of fatty acid-enabled resistance to AFN-1252. Ten parallel cultures of S. aureus Δ*agrA* were exposed to 3 rounds of daptomycin (20 μg ml^−1^) and AFN-1252 (0.15 μg ml^−1^) in the absence or presence of fatty acid cocktail and the absence or presence of human serum albumin (HSA) before bacterial survival (A), the AFN-1252 MICs (B, C, D), as well as the daptomycin MICs (E, F, G) were determined in the absence or presence of fatty acids after each round of exposure to the antibiotic combination. Each symbol represents an independent culture (*n* = 10 in each case). Differences in survival between rounds of antibiotic exposure under identical conditions were identified using a one-way ANOVA with Dunn’s multiple-comparison test (*, *P* < 0.001).

Having confirmed that AFN-1252 resistance can arise in the presence of fatty acids, the next objective was to test whether combination therapy with daptomycin could prevent this. As expected from previous data (Fig. 4), bacterial killing with daptomycin–AFN-1252 combination therapy was highly effective for the first two exposures, where bacterial survival was 1% or less after 8 h. An increase in bacterial survival was observed on the third exposure, but bacterial growth was still inhibited, with CFU counts not exceeding the count in the original inoculum (Fig. 9A). Furthermore, this increase in survival was independent of the presence of fatty acids (Fig. 9A).

In contrast to experiments with AFN-1252 alone, repeated exposure of S. aureus to AFN-1252 in the presence of daptomycin did not lead to an increase in the MIC of the FASII inhibitor, even in the presence of fatty acids (Fig. 9B to D), nor was there any increase in the daptomycin MIC (Fig. 9E to G). Together, these data demonstrate that daptomycin prevented the emergence of fatty acid-dependent resistance to AFN-1252 when the two antibiotics were used in combination.

Despite the increase in bacterial survival on the third exposure, the CFU counts did not exceed the count in the original inoculum (Fig. 9A), and the unchanged MIC values (Fig. 9B to G) indicated that AFN-1252 and daptomycin still had bacteriostatic activity (i.e., while the antibiotics did not cause a drop in CFU counts, they still prevented bacterial replication).

### AFN-1252 blocks daptomycin-induced phospholipid release in AFN-1252-resistant strains.

Having established that the combination of daptomycin and AFN-1252 prevented the emergence of AFN-1252 resistance, we next wanted to understand the underlying mechanism.

**FIG 10:**
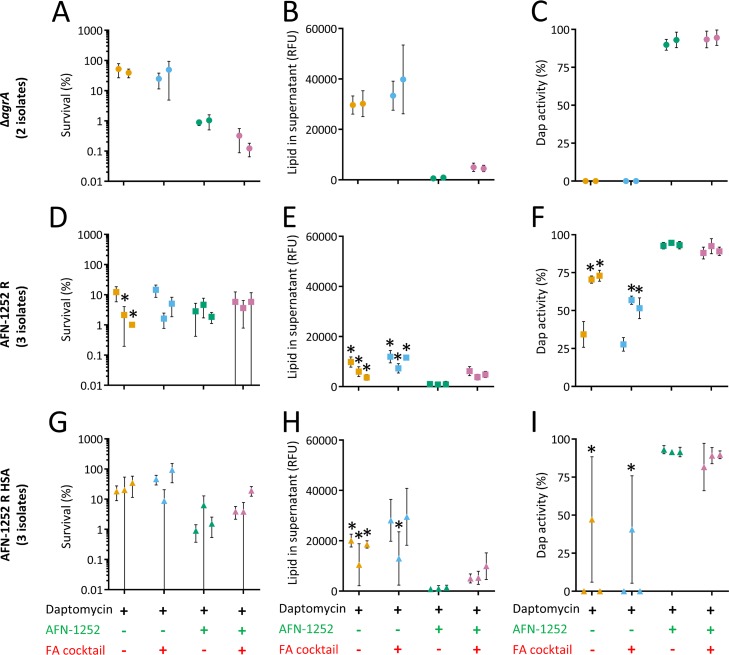
AFN-1252 prevents daptomycin-induced phospholipid release, even in the case of AFN-1252-resistant strains. Two independent isolates (each represented by an individual circle) of the S. aureus Δ*agrA* mutant (Δ*agrA*) that had not been exposed to antibiotic (A, B, C), three independent isolates of the S. aureus Δ*agrA* mutant (each represented by an individual square) that had acquired resistance to AFN-1252 in the presence of the fatty acid cocktail but the absence of HSA (AFN-1252 R) (D, E, F), or three independent isolates of the S. aureus Δ*agrA* mutant (each represented by an individual triangle) that had acquired resistance to AFN-1252 in the presence of the fatty acid cocktail and HSA (AFN-1252 R HSA) (G, H, I) were exposed to daptomycin (Dap) in the presence or absence of various combinations of AFN-1252 and fatty acid (FA) cocktail for 8 h. After this time, bacterial survival (A, D, G), the quantity of released phospholipid (B, E, H), and the activity of daptomycin (C, F, I) were determined. Data represent the means from 3 independent experiments, and error bars represent the standard deviation of the mean. Differences in survival, phospholipid release, or daptomycin activity were compared between the AFN-1252-susceptible USA300 Δ*agrA* isolates and AFN-1252-resistant isolates using a one-way ANOVA with Dunn’s multiple-comparison test (*, *P* < 0.01).

As described above (Fig. 4), two independent colony picks of the Δ*agrA* mutant that had not previously been exposed to antibiotics survived exposure to daptomycin by releasing phospholipids that completely inactivated the antibiotic (Fig. 10A to C). However, the presence of AFN-1252 increased the bactericidal activity of daptomycin by preventing phospholipid release and, thus, preserving the activity of the lipopeptide antibiotic, regardless of the presence of fatty acids (Fig. 10A to C).

Next, we assessed the survival of bacteria from 3 independent cultures that had acquired resistance to AFN-1252 during exposure to the antibiotic in the presence of fatty acids but not HSA (AFN-1252 R). Of these 3 isolates, 2 were more susceptible to daptomycin than the Δ*agrA* mutant, apparently because they released lower levels of phospholipids that failed to fully inactivate the lipopeptide antibiotic (Fig. 10D to F). The remaining isolate reduced daptomycin activity by 70%, explaining its enhanced survival in the presence of daptomycin relative to that of the other 2 isolates. However, the presence of AFN-1252 completely abolished the ability of any of these isolates to inactivate daptomycin, even when exogenous fatty acids were present (Fig. 10D to F).

We then examined S. aureus isolates from 3 independent cultures that had acquired resistance to AFN-1252 during exposure to the antibiotic in the presence of fatty acids and HSA (AFN-1252 R HSA). The survival of these three AFN-1252-resistant isolates after exposure to daptomycin alone was not significantly lower than that seen for the AFN-1252-sensitive Δ*agrA* mutant. This was due to the release of sufficient phospholipid to inactivate all or most of the daptomycin that the bacteria were incubated with (Fig. 10G to I). However, despite the ability of these bacteria to grow in the presence of AFN-1252 when exogenous fatty acids were available, the FASII inhibitor almost completely blocked daptomycin-induced phospholipid release from all three isolates, even when the fatty acid cocktail was present (Fig. 10G to I).

Together, these data reveal that fatty acid-enabled AFN-1252 resistance results in a reduced ability to release phospholipids in response to daptomycin alone (Fig. 10E and H). Furthermore, although these strains were deemed resistant to AFN-1252, daptomycin-induced phospholipid release was inhibited by the FASII inhibitor, even in the presence of exogenous fatty acids (Fig. 10E and H). This provides additional evidence that daptomycin-induced phospholipid release is dependent upon endogenous, FASII-mediated fatty acid biosynthesis and that utilization of exogenous fatty acids to bypass FASII for lipid synthesis does not enable daptomycin-induced phospholipid release. As such, daptomycin-induced phospholipid release is efficiently blocked by AFN-1252, preventing inactivation of the lipopeptide antibiotic.

## DISCUSSION

The high rate of failure of daptomycin treatment for osteomyelitis and complicated skin infections caused by S. aureus warrants efforts to understand the determinants of therapeutic outcomes and identify new approaches to enhance bacterial clearance (8). In agreement with our previous work (9, 10) and that of others (31), the data presented here revealed that S. aureus at a high density can inactivate daptomycin, which promotes the survival of bacteria exposed to this antibiotic. Subsequent *in vitro* studies revealed that the FASII inhibitor AFN-1252 prevents the inactivation of daptomycin by clinical S. aureus isolates, while daptomycin reduces the emergence of spontaneous fatty acid-dependent resistance to the FASII inhibitor, at least for the USA300 strain examined here.

It is increasingly clear that the host environment modulates the susceptibility of bacterial pathogens to antibiotics due to the scarcity of nutrients and the induction of stress responses that result in changes in bacterial physiology (32, 33). Serum contains high concentrations of fatty acids, which can be exploited by S. aureus to produce phospholipids, reducing the metabolic costs associated with membrane biogenesis (21, 23). In keeping with this, we found that the presence of specific exogenous fatty acids, such as oleic or lauric acid, enhanced phospholipid release in response to daptomycin. However, S. aureus has strict requirements for the type of fatty acids that it can incorporate, and, at least for wild-type strains, each phospholipid must have at least one fatty acid tail synthesized endogenously via FASII (33). This requirement for FASII-mediated fatty acid biosynthesis to generate phospholipids was underlined by the ability of AFN-1252 to completely block phospholipid decoy release, regardless of the presence of oleic acid (34). This provides evidence that daptomycin–AFN-1252 combination therapy may not be compromised by the availability of fatty acids in the host.

While some exogenous fatty acids can be used for phospholipid biosynthesis during staphylococcal growth, it appears that their contribution to daptomycin-induced phospholipid release is severely compromised by the presence of serum albumin, which sequesters the fatty acids (28). As described above, there is clear evidence that S. aureus can partially substitute endogenous fatty acid biosynthesis for exogenous host-derived fatty acids in the generation of phospholipids. However, our data demonstrate that the presence of serum albumin reduces the efficiency of this process sufficiently to prevent their use in daptomycin-induced phospholipid release, which must occur quickly if the bacteria are to survive exposure to the rapidly bactericidal antibiotic.

In addition to providing nutrients, the host environment can also modulate bacterial signaling systems and virulence factor production. We have shown previously that the inactivation of daptomycin by released phospholipids is inhibited by the concomitant production of PSMα peptides in response to activation of the Agr quorum-sensing system (9). However, serum blocks Agr signaling and sequesters PSMs (14–17), which explains why both the Agr-competent wild-type strain and the Δ*agrA* mutant were able to inactivate daptomycin when experiments were conducted in human serum. Similarly, the Agr status of the clinical isolates had no impact on their ability to inactivate daptomycin in the presence of serum, in contrast to what we have seen previously in TSB alone (9).

The mechanism by which S. aureus releases phospholipids in response to daptomycin is unknown. However, the finding that a majority, but not all, clinical isolates can inactivate daptomycin suggests that it may be possible to identify the genetic determinants of phospholipid release by whole-genome sequencing of clinical isolates and subsequent genome-wide association studies. Clearly, the release of phospholipids that can inactivate daptomycin occurs via an active process (9, 10). It appears that this system functions most efficiently at lower concentrations of daptomycin, since higher concentrations of the antibiotic presumably kill the bacteria before they can synthesize and release the phospholipids. This may explain why the efficacy of daptomycin is greater at a higher therapeutic dose of the antibiotic, particularly for endocarditis (8). Unfortunately, the toxicity of the lipopeptide antibiotic limits the concentration that can be used to treat infection (1). Therefore, the finding that the bactericidal activity of low concentrations of daptomycin can be promoted by AFN-1252, even in the presence of serum, may have clinical value as a route to improving patient outcomes.

The successful clinical development of AFN-1252 would be a welcome addition to the arsenal of antistaphylococcal antibiotics. However, although wild-type bacteria are dependent upon the endogenous FASII pathway to generate fatty acids for phospholipid biosynthesis, our data provide additional evidence that this is not the case in strains that have acquired mutations within the *fabD* lipid biosynthetic gene loci (25, 26). These mutants can bypass FASII-mediated fatty acid production, conferring resistance to AFN-1252 in the presence of exogenous fatty acids (25, 26). It has been suggested that FASII bypass could compromise the long-term therapeutic viability of FASII inhibitors, such as AFN-1252, a view that is supported by the identification of clinical isolates that are able to resist AFN-1252 in the presence of exogenous fatty acids (25). Despite this, early clinical studies have shown that AFN-1252 can successfully be used to treat skin and soft tissue infections, albeit in a relatively small number of patients (18). Furthermore, a study using a murine thigh infection model suggested that AFN-1252 is efficacious for the treatment of deep-seated infections, where host-derived fatty acids are likely to be available to S. aureus (35). Therefore, it remains to be seen whether resistance to AFN-1252 becomes a significant clinical problem. However, given the ability of S. aureus to rapidly acquire resistance to antibiotics, it seems prudent to develop therapeutic strategies to prevent or overcome the emergence of resistance to AFN-1252. Our data provide support for the concept of spontaneous AFN-1252 resistance development via fatty acid-dependent FASII bypass, but they also demonstrate that the frequency at which resistance emerges can be significantly reduced by the presence of daptomycin, at least *in vitro*.

The combination of AFN-1252 and daptomycin could be described as a mutually beneficial pairing; while AFN-1252 promotes daptomycin activity by blocking phospholipid release, daptomycin enhances AFN-1252 efficacy by preventing the emergence of resistance. This finding contributes to our growing appreciation for the potential of combination therapy approaches to circumvent resistance mechanisms. A well-established example of this is the combination of daptomycin and β-lactams that target penicillin-binding protein 1 (PBP1). The mechanisms responsible are complex and not fully defined. However, daptomycin increases the expression of *pbpA*, which appears to be important to enable the bacterium to survive exposure to the lipopeptide (36, 37). Blockage of PBP1 function therefore promotes daptomycin activity against S. aureus, possibly via the increased binding of the lipopeptide antibiotic to the bacterial membrane (36, 37). In turn, daptomycin reduces the quantity of PBP2a available, which reduces the resistance of S. aureus to β-lactams (38, 39). This phenomenon, known as the seesaw effect, significantly promotes the killing of S. aureus relative to that by each of the antibiotics individually and is currently being assessed as a therapeutic option in a clinical trial (40).

In summary, the presence of AFN-1252 prevented the phospholipid-mediated inactivation of daptomycin by clinical MRSA isolates, while daptomycin inhibited the fatty-acid dependent emergence of resistance to AFN-1252. Therefore, we propose that the combination of AFN-1252 and daptomycin may have therapeutic value for the treatment of serious MRSA infections.

## MATERIALS AND METHODS

### Bacterial strains and growth conditions.

Staphylococcus aureus USA300 wild-type and Δ*agrA* mutant strains (9) or clinical isolates (Table 1) were grown in tryptic soy broth (TSB) or on tryptic soy agar (TSA). For some assays TSB was supplemented with fatty acids, including oleic acid, linoleic acid, palmitic acid, myristic acid, or lauric acid (all were obtained from Sigma-Aldrich). Since the serum concentrations of these fatty acids vary from 2 μM (lauric acid) to 122 μM (oleic acid) (27), assays were initially done with a single concentration (20 μM) within this range, although some assays with oleic acid used up to 100 μM of the fatty acid. For some assays, HSA was included (10 mg ml^−1^) to sequester fatty acids (22). Some assays used TSB containing 0.5 mM MgCl_2_ and 1.25 mM CaCl_2_ supplemented with 50% normal human serum (type AB positive; Sigma-Aldrich) to mimic the host environment. Bacteria inoculated onto TSA plates were incubated statically at 37°C for 15 to 17 h in air unless otherwise stated. Clinical isolates were also plated onto Columbia blood agar (CBA) containing 5% sheep’s blood to enable assessment of hemolysis, which is a useful proxy for Agr activity (9). Liquid cultures were grown in 3 ml broth in 30-ml universal tubes by suspending a single colony from TSA plates and incubated at 37°C with shaking at 180 rpm to facilitate aeration for 15 to 17 h to stationary phase. Staphylococcal CFU were enumerated by serial dilution in sterile phosphate-buffered saline (PBS) and plating of aliquots onto TSA. Bacterial stocks were stored in growth medium containing 20% glycerol at −80°C.

### Antibiotic killing kinetics.

S. aureus was grown to stationary phase in 3 ml TSB with shaking (180 rpm) at 37°C in 30-ml universal tubes as described above. Bacteria were subsequently adjusted to a concentration of ∼1 × 10^8^ bacteria ml^−1^ in fresh TSB containing 0.5 mM CaCl_2_ to maintain consistency with previous work from our group (9) and others (41) in resistance emergence assays, before antibiotics were added at the following concentrations: daptomycin at 20 μg ml^−1^ (Tocris) and AFN-1252 at 0.15 μg ml^−1^ (MedchemExpress). For some experiments, TSB was supplemented with 50% normal human serum (Sigma-Aldrich), human serum albumin, or fatty acids, as indicated above. For assays with 50% normal human serum, the TSB component was supplemented with 0.5 mM MgCl_2_ and 1.25 mM CaCl_2_ to provide physiological concentrations. Cultures were then incubated at 37°C with shaking (180 rpm), and bacterial viability was determined by the use of CFU counts from samples taken every 2 h for 8 h.

### Daptomycin activity determination.

The activity of daptomycin during incubation with S. aureus was quantified as described previously (9, 10). A well of 10 mm was made in TSA plates containing 0.5 mM CaCl_2_, followed by the spreading of stationary-phase wild-type strain USA300 (60 μl, ∼10^6^ ml^−1^ in TSB) across the surface. When AFN-1252 was used in the assays, TSA was spread with Streptococcus agalactiae COH1 instead of S. aureus, as this bacterium is naturally resistant to the FASII inhibitor but susceptible to daptomycin. Thereafter the plate was dried before the wells were filled with filter-sterilized culture supernatant. The plates were then incubated for 16 h at 37°C before the zone of growth inhibition around the well was measured at 4 perpendicular points. To accurately quantify daptomycin activity, a standard plot was generated for the zone of growth inhibition around wells that were filled with TSB supplemented with a range of daptomycin concentrations. This enabled the conversion of the size of the zone of inhibition into percent daptomycin activity.

### Phospholipid detection and quantification.

S. aureus membrane lipid was detected and quantified using the FM-4-64 dye (Life Technologies) as described previously (9, 10). Bacterial culture supernatants (200 μl) were recovered by centrifugation (17,000 × *g*, 5 min) and then mixed with FM-4-64 dye to a final concentration of 5 μg ml^−1^ in the wells of clear flat-bottom microtiter plates with black walls appropriate for fluorescence readings (Greiner Bio-One). Fluorescence was measured using a Tecan microplate reader, with excitation at 565 nm and emission at 660 nm being used to generate values expressed as relative fluorescence units (RFU). Samples were measured in triplicate for each biological repeat. TSB with or without fatty acids was mixed with the FM-4-64 dye and used as a blank. The readings were analyzed by subtracting the values from the blank readings and plotted against time.

### Antibiotic resistance selection assay.

Stationary-phase S. aureus was inoculated at ∼10^8^ CFU ml^−1^ into 3 ml TSB with 0.5 mM CaCl_2_ containing antibiotics, as specified above, for 8 h per exposure. Daptomycin (20 μg ml^−1^) and AFN-1252 (0.15 μg ml^−1^) were used singly or in combination. After 8 h, bacterial survival was determined by calculating the fold change (for assays with the bacteriostatic AFN-1252 only) or the percent change (for assays with the bactericidal antibiotic daptomycin) in the number of CFU relative to that in the inoculum. For repeated antibiotic exposure, 1 ml was removed from each culture after antibiotic exposure and centrifuged (3 min, 17,000 × *g*), and the resulting pellet was washed once in TSB before resuspension in 100 μl TSB. This was used to inoculate 3 ml TSB before incubation for 16 h at 37°C with shaking (180 rpm) in the absence of antibiotics. Bacterial exposure to antibiotics was then repeated twice for a total of three repeated exposures. In some experiments, the broth was supplemented with a fatty acid cocktail prepared as follows: myristic, palmitic, and oleic acids (all from Sigma-Aldrich) were made up to 100 mM in dimethyl sulfoxide (DMSO) as described previously (26). Where used, the fatty acid cocktail was diluted 1 in 2,000 in culture medium to obtain a final concentration of 50 μM to provide a balance between previous work (26) and physiological relevance (27). In some cases, TSB was also supplemented with human serum albumin (Sigma-Aldrich) at 10 μg ml^−1^ to improve the solubility of the fatty acids without reducing the activity of the antibiotics.

### Determination of antibiotic MICs.

Antibiotic susceptibility was determined using the broth microdilution procedure as described previously (42) to generate MICs for daptomycin and AFN-1252. Antibiotics were serially diluted in 2-fold steps in culture medium in a 96-well microtiter plate to obtain a range of concentrations. In some assays, a fatty acid cocktail (50 μM) was added to the broth as described above for the resistance selection assay. Stationary-phase bacteria were added to the wells to give a final concentration of 5 × 10^5^ CFU ml^−1^, and the microtiter plates were incubated statically in air at 37°C for 18 h. The MIC was defined as the minimum concentration of antibiotic needed to inhibit the visible growth of the bacteria (38). For some assays, the fold change in the MIC relative to the MIC of the USA300 Δ*agrA* mutant which had not been exposed to antibiotics was calculated.

### PCR amplification and sequencing of *fabD*.

PCR amplification of *fabD* was performed using the colony PCR technique. A single colony was suspended in 50 μl nuclease-free H_2_O, microwaved for 3 min to lyse the cells, and then centrifuged for 2 min (13,300 rpm, room temperature) to pellet the cell debris. Supernatant (5 μl) containing genomic DNA was used for each PCR, in which Phusion high-fidelity DNA polymerase was used (New England Biolabs). PCR cycling conditions were as follows: 98°C for 10 min and 30 cycles of 98°C for 30 s, 56.8°C for 30 s, and 72°C for 30 s, with a final step at 72°C for 5 min. PCR products were purified using a QIAquick PCR purification kit (Qiagen) following the manufacturer’s instructions. Purified DNA was sequenced by Sanger sequencing (GATC Biotech, Germany) using the same forward primer used for PCR amplification. Primer sequences were obtained from reference 26 and were as follows: 5′-GAAGGTACTGTAGTTAAAGCACACG-3′ for primer FabDfd and 5′-GCTTTGATTTCTTCGACTACTGCTT-3′ for primer FabDrev.
